# Beverage consumption patterns among 4–19 y old children in 2009–14 NHANES show that the milk and 100% juice pattern is associated with better diets

**DOI:** 10.1186/s12937-018-0363-9

**Published:** 2018-05-24

**Authors:** Matthieu Maillot, Colin D. Rehm, Florent Vieux, Chelsea M. Rose, Adam Drewnowski

**Affiliations:** 1 MS-Nutrition, 27 bld Jean Moulin Faculté de Médecine la Timone, Laboratoire NORT, 13385 Marseille cedex 5, France; 2 Albert Einstein College of Medicine, Montefiore Medical Center, New York, NY 10467 USA; 3 Center for Public Health Nutrition, University of Washington, Box 353410, Seattle, WA 98195 USA

**Keywords:** Milk, 100% juice, Beverage consumption, Children, Diet quality

## Abstract

**Background:**

Patterns of beverage consumption among children and adolescents can be indicative of food choices and total diet quality.

**Methods:**

Analyses of beverage consumption patterns among 8119 children aged 4–19 y were based on the first 24-h recall of the National Health and Nutrition Examination Survey (2009–14 NHANES). Four pre-defined beverage patterns were: 1) milk pattern; 2) 100% juice pattern; 3) milk and 100% juice pattern; and 4) other caloric beverages. Food- and nutrient-based diet quality measures included the Healthy Eating Index 2010.

**Results:**

Most children drank other caloric beverages, as opposed to milk (17.8%), 100% juice (5.6%), or milk and 100% juice (13.5%). Drinkers of milk and 100% juice had diets that did not differ from each other in total calories, total and added sugars, fiber, or vitamin E. Milk drinkers consumed more dairy and had higher intakes of calcium, potassium, vitamin A and vitamin D as compared to all other patterns. Juice drinkers consumed more total fruit, same amounts of whole fruit, and had higher intakes of vitamin C as compared to the other consumption patterns. Drinkers of both milk and 100% juice had the highest HEI 2010 scores of all the consumption patterns.

**Conclusions:**

Beverage consumption patterns built around milk and/or 100% juice were relatively uncommon. Promoting the drinking of milk and 100% juice, in preference to other caloric beverages, may be an effective strategy to improve children’s diet quality. Restricting milk and 100% juice consumption may encourage the selection of other caloric beverages.

**Electronic supplementary material:**

The online version of this article (10.1186/s12937-018-0363-9) contains supplementary material, which is available to authorized users.

## Background

The place of milk and 100% fruit juice in U.S. children’s diets continues to be a topic of debate [1]. The consumption of whole milk and 100% fruit juice by young children has been linked to higher body weight in some studies [2, 3], though not in others [4, 5]. To prevent excess weight gain, the American Academy of Pediatrics (AAP) recommends that children switch to low-fat or non-fat milk after the age of 2 y [6]. The AAP has also set limits on the consumption of 100% juices. The suggested amounts are 4–6 oz./d for children aged 4–6 y and up to 8 oz./d for children aged 7–18 y [7].

The current trend in nutritional epidemiology is to examine food and beverage patterns as opposed to individual nutrients, foods, or dietary ingredients [8–12]. Most published studies have explored children’s beverage consumption, a dependent variable, by gender, age subgroup, household income, or race/ethnicity [13–15]. Classifying children by their beverage consumption patterns is a relatively novel approach.

The present analyses, based on the three most recent cycles of the National Health and Nutrition Examination Survey (NHANES 2009–2014), identified children aged 4–19 y whose beverage drinking patterns were built around 1) milk and milk beverages, 2) 100% juices, and 3) milk and 100% juices. Their diet quality was then compared to that of children who drank other caloric beverages in preference to milk or 100% juice.

The primary hypothesis was that beverage drinking patterns characterized by milk and/or 100% juice would be associated with better dietary choices and higher quality diets. The secondary hypothesis was that beverage patterns featuring both milk and 100% juice would be associated with best dietary nutritional profiles and with highest Healthy Eating Index (HEI 2010) scores, a composite measure of diet quality.

## Methods

### The 2009–14 NHANES sample

Dietary intake data came from 3 cycles of the National Health and Nutrition Examination Survey (2009–10, 2011–12 and 2013–14 NHANES). Data from 2013 to 2014 NHANES were released in October 2016. Data analyses, based on the first-day single 24-h recall, were conducted for the entire population (4–19 y, *n* = 8119) and separately by age group: 4–8 y, 9–13 y, and 14–19 y.

### Dietary intake assessment

The NHANES 24-h recall multi-pass method, conducted by a trained interviewer [16, 17], captured types and amounts of all food and beverages consumed during the previous 24-h, along with the time and occasion for each food item. For children aged 4–5 y the dietary recall was completed by a proxy respondent (parent or guardian). For children 6–11 y, the child was the primary respondent, but a proxy respondent was present and able to assist. Children aged 12–19 y were the primary respondents, but could be assisted by an adult when needed.

### Classification of beverages

Beverages were classified into 14 categories following the USDA coding scheme: milk and milk beverages, citrus juices, apple juices (including fruit juice blends), other non-citrus juices, soda (regular & diet), fruit drinks (regular & diet), sports and energy drinks (regular & diet), vegetable juice, water (bottled & tap), flavored and enhanced water, alcoholic beverages, coffee, tea, and meal replacement beverages (See Additional file 1: Table S1).

The milk category included whole milk, 2%, 1%, skim, flavored milk, and nutritionally equivalent milk alternatives, but excluded milkshakes, eggnog, and Yoo-Hoo type milk drinks. Milk consumers were defined as those consuming milk as a beverage, and not e.g. with cereal, using NHANES food combination codes.

The 100% juices included citrus juices, apple juice, and non-citrus juices, and vegetable juices. The 100% fruit juice blends (e.g. apple-cranberry) were included but sweetened fruit-based drinks with added sugars were not. All the remaining beverages were classified as “other.” Drinking water (bottled and tap) was excluded, as were alcohol and meal replacements.

### Classification of beverage consumption patterns

Children can consume multiple beverages within a 24-h period. Beverage consumption patterns were categorized into 4 groups 1) milk and milk beverages pattern (no 100% juice); 2) 100% juice pattern (no milk); 3) milk and 100% juice pattern; and 4) other beverages pattern. To be assigned to the milk and/or 100% juice patterns, the consumption of milk and/or 100% juice (separate or joint) had to exceed the consumption of all other caloric beverages. A similar method had been used to identify drinkers of regular and diet sodas [18]. The groups were characterized as 1) milk drinkers; 2) 100% juice drinkers; 3) milk and 100% juice drinkers and 4) drinkers of other caloric beverages. Given the widespread use of caloric beverages, we expected the milk and/or 100% juice beverage patterns to be relatively infrequent.

### Analytic approach

Dietary outcomes of interest were: dietary energy (kcal), total sugars (grams), added sugars (teaspoon equivalents), calcium (mg), vitamin D (mcg), vitamin A (IU), vitamin E (mcg), vitamin C (mg), potassium (mg), and fiber (g). Food groups of interest were dairy products (milk, yogurts and cheese), total fruit (whole fruit and fruit juice) and whole fruit, and total vegetables. The Healthy Eating Index 2010 (HEI 2010) was the principal measure of diet quality. The HEI measures compliance with the Dietary Guidelines for Americans along a 100-point scale and food group components are represented as “cup-equivalents.” For example, 1 cup-equivalent of milk or juice equals 1 cup (237 ml).

All analyses accounted for the complex survey design of NHANES data and are representative of the US population. Data analyses were conducted using Stata 13.1 (College Station, TX) and SAS 9.4 (SAS institute, Cary, NC).

### Funding

Funding for these analyses of publicly available federal data was provided by the Dr. Pepper Snapple group.

## Results

Four out of five children (82.0%) drank multiple beverages on the same day. Out of 8119 children, 1209 (17.8%) showed the milk pattern; 543 (5.6%) showed the 100% juice pattern, and 1081 (13.5%) showed the milk and 100% juice pattern. The majority of children (5286 or 63.2%) drank other caloric beverages, with smaller amounts of milk, juice, or both.

### Beverage patterns

The NHANES sample was evenly split between boys (4160 or 51.0%) and girls (3959 or 49.0%). Beverage consumption patterns varied by gender (*p* = 0.0008) and age group (*p*< 0.001). The milk pattern was more common among boys than among girls (54.1 vs. 45.9%); the 100% juice pattern was more common among girls than among boys (57.7 vs. 42.3%), consistent with past studies [19–21]. Other caloric beverages were consumed equally by both boys and girls (49.6% male vs. 50.4% female). The prevalence of the milk and 100% juice pattern dropped precipitously with age (from 60.8 to 14.3%), whereas the consumption of other caloric beverages increased (21.4 to 48.0%), doubling from the 4–8 y to the 14–19 y age group.

Milk drinkers drank 511.4 g of milk (95% CI: 478.8, 544.1) and 151.2 g of other beverages (95% CI: 127.5, 174.9), but no 100% juice. Juice drinkers drank 404.3 g of juice (95% CI: 365.7, 442.9) and 102.8 g of other beverages (95% CI: 78.4, 127.1) but no milk. Milk and juice drinkers drank 376.5 g of milk (95% CI: 350.4, 402.5), 274.5 g of juice (95% CI: 258.2, 290.7) and 155.2 g of other beverages (95% CI: 135.9, 174.4). Most children (63%) drank large volumes of other beverages (758.7 g (95% CI:729.8, 787.6)) but negligible amounts of either milk (89.3 g (95% CI: 80.7, 98.0)) or 100% juice (38.2 g (95%CI: 31.4, 45.1)).

Milk drinkers derived 15.3% of energy from milk; juice drinkers derived 11.6% energy from 100% juice. Drinkers of milk and juice derived 17.6% energy from those two beverages combined. No significant relation with age was observed. By contrast the amounts of other caloric beverages increased sharply with age: from 11.8% energy in young children to 15.1% energy in adolescents. Children who drank 100% juice had energy intakes that were lower when compared to drinkers of other beverages.

Drinkers of milk and 100% juice consumed amounts of 100% juice that were close to AAP guidelines: 250 g for 4–8 y (95% CI: 231.4, 268.9), 270 g for 9–13 y (95% CI: 253.0, 285.6) and 386 g for 14–19 y (95% CI: 313.5, 460.1). The age-related increase in consumption was modest for milk drinkers (milk 457 to 610 g, p for trend < 0.001) and for 100% juice drinkers (FJ 346 to 477 g, p for trend = 0.007), but very sharp for other beverages (502 to 950 g, p for trend < 0.001).

### Diet quality among the beverage patterns

Table 1 shows that children classified as milk drinkers had diets with 3.44 cup-eq/day of dairy, largely from milk (2.53 cup-eq). Their consumption of milk (but not yogurt or cheese) was higher compared to 100% juice drinkers (0.47 cup-eq, *p* < 0.001) or drinkers of other beverages (0.83 cup-eq, *p* < 0.001).

**Table 1:** Food patterns, energy, and selected nutrients for drinkers of milk, 100% juice, milk and 100% juice, and other beverages NHANES 2011–2014: Ages 4-19 y [*n* = 8119]

	Milk Pattern			100% Juice Pattern			Milk and 100% Juice Pattern			Other Beverages Pattern			*p* ^a^	*Ad* ^b^
	*n* = 1209			*n* = 543			*n* = 1081			*n* = 5286				
	Mean	95% CI		Mean	95% CI		Mean	95% CI		Mean	95% CI			
Food groups and HEI														
Total Dairy (cup-eq)	3.44	3.25	3.63	1.30	1.13	1.48	2.85	2.69	3.01	1.76	1.70	1.83	*<.001*	*<.001*
Milk (cup-eq)	2.53	2.39	2.67	0.47	0.42	0.52	2.00	1.87	2.13	0.83	0.79	0.87	*<.001*	*<.001*
Yogurts (cup-eq)	0.05	0.03	0.06	0.04	0.02	0.06	0.09	0.06	0.12	0.04	0.03	0.05	*0.007*	*0.012*
Cheese (cup-eq)	0.84	0.74	0.94	0.78	0.62	0.94	0.75	0.65	0.84	0.88	0.83	0.93	*0.034*	*0.120*
Total Fruits (cup-eq)	0.72	0.65	0.80	2.31	2.15	2.48	2.02	1.92	2.12	0.82	0.74	0.89	*<.001*	*<.001*
Whole Fruits (cup-eq)	0.70	0.62	0.77	0.68	0.55	0.81	0.89	0.81	0.97	0.60	0.54	0.67	*<.001*	*0.001*
Total vegetable (cup-eq)	0.91	0.81	1.00	0.86	0.75	0.96	0.91	0.82	1.00	0.99	0.95	1.03	*0.034*	*0.33*
HEI 2010	49.65	48.79	50.51	52.44	51.09	53.79	55.70	54.36	57.04	44.36	43.69	45.02	*<.001*	*<.001*
Energy and nutrients														
Energy (kcal)	1924	1828	2020	1798	1718	1879	1998	1933	2063	2029	1991	2066	*<.001*	*–*
Total sugars (g)	109	104	113	109	103	115	133	129	137	130	127	134	*<.001*	*<.001*
Added sugars (teaspoon eq)	13	13	14	11	10	12	14	13	15	23	22	24	*<.001*	*<.001*
Fiber (g)	15	14	16	15	14	15	15	15	16	14	14	14	*0.008*	*<.001*
Calcium (mg)	1379	1308	1450	920	837	1004	1314	1262	1366	934	909	958	*<.001*	*<.001*
Potassium (mg)	2475	2339	2611	2182	2069	2295	2713	2629	2796	2100	2058	2142	*<.001*	*<.001*
Vitamin A (IU)	796	753	839	499	461	538	698	660	736	527	510	545	*<.001*	*<.001*
Vitamin C (mg)	47	40	54	144	130	158	118	111	124	70	66	75	*<.001*	*<.001*
Vitamin D (mcg)	9	9	10	4	3	4	8	8	9	4	4	4	*<.001*	*<.001*
Vitamin E (mcg)	7	6	8	7	6	7	7	6	7	7	7	8	*0.032*	*0.008*

^a^unadjusted regression models

^b^regression models adjusted for gender, poverty level and total dietary energy

Children classified as 100% juice drinkers had diets with 2.31 cup-eq of total fruit (including 0.68 cup-eq whole fruit). Importantly, their diets were not reduced in whole fruit. Milk drinkers and juice drinkers ate comparable amounts of vegetables (*p* = 0.999).

Drinkers of milk and 100% juice had diets with 2.85 cup-eq of dairy, 2.02 cup-eq of total fruit, including 0.89 cup-eq of whole fruit. This group also had the highest HEI 2010 scores (55.70), compared to juice drinkers (52.44, *p* = 0.005), milk drinkers (49.65, *p* < 0.001) and to children who drank neither milk nor 100% juice (44.36, *p* < 0.001).

**Fig. 1:**
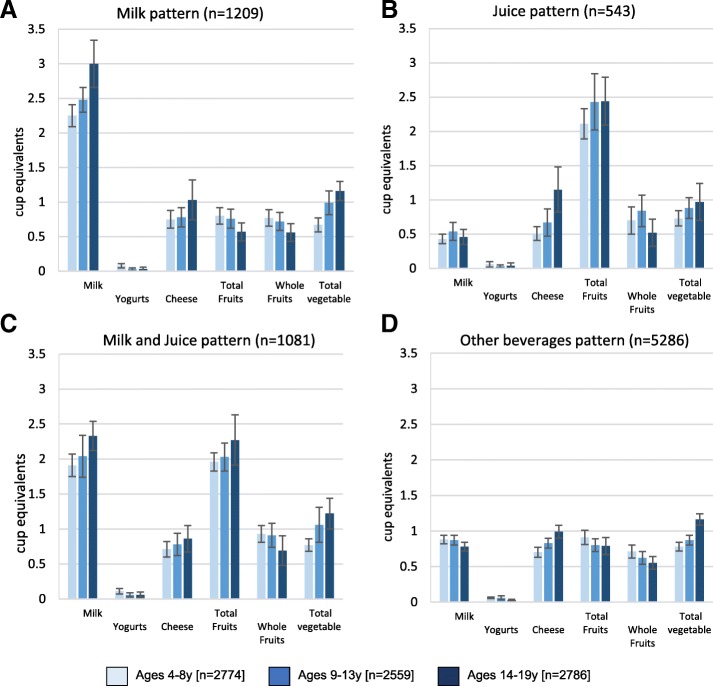
**a**-**d**: Food group intake by beverage pattern and age group, NHANES 2011–2014

Figure 1 shows that comparable effects were observed across all age groups. Drinkers of milk, 100% juice or both had higher quality diets as compared to drinkers of other caloric beverages. Drinkers of both milk and 100% juice had the highest HEI 2010 scores.

Table 1 also shows that there were no significant differences between milk and 100% juice drinkers in terms of total energy (1924 vs. 1798 kcal/d, *p* = 0.241); total sugars (108.6 vs. 109.3 g, *p* = 0.153); added sugars (13.4 vs. 11.0 tsp-eq, *p* = 0.250), or fiber (14.8 vs. 14.5 g, *p* = 0.999). Milk drinkers consumed more calcium (1379 mg vs 920 mg), vitamin A (792 vs. 499 IU, *p* < 0.001) and vitamin D (9.1 vs. 3.9 mcg, *p* < 0.001) than did juice drinkers. Diets of juice drinkers were higher in vitamin C (144 vs. 47 mg, *p* < 0.001). There were no differences in vitamin E (*p* = 0.999).

Children classified as drinkers of milk and 100% juice had more nutrient-rich diets than did drinkers of either milk or 100% juice alone. Their potassium intakes were higher than for the other 3 beverage patterns (*p* < 0.001), calcium was higher than for juice (*p* < 0.001) or for other beverages (*p* < 0.001). Intakes of calcium (*p* < 0.029), vitamin A (*p* < 0.001), and vitamin D (*p* < 0.001) were higher than for milk drinkers. The diet’s content of vitamin C (118 mg/d) was higher than for milk drinkers (47 mg/d, *p* < 0.001) but not as high as for juice (144 mg/d, *p* < 0.001). Drinkers of other beverages had the highest-energy diets with most added sugar.

HEI 2010 scores were highest for the milk and 100% juice pattern (55.70), followed by the juice pattern (52.44), and then by milk pattern (49.65) and other beverages (44.36).

**Fig. 2:**
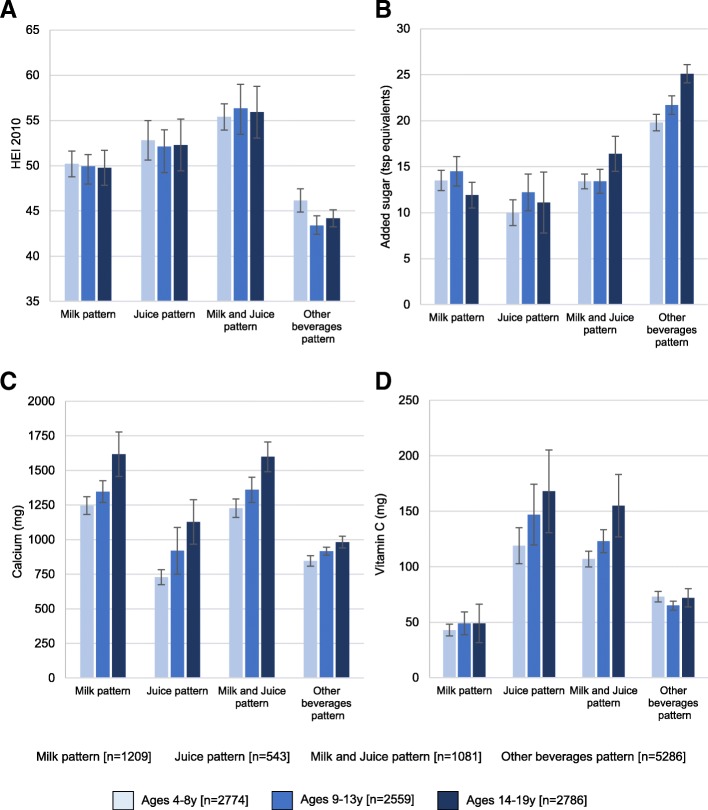
**a**-**d**: HEI and nutrient intakes by beverage pattern and by age group, NHANES 2011–2014

Figure 2 shows that the drinkers of milk and 100% juice had the highest HEI 2010 scores. Whereas milk-based patterns provided more calcium, juice based patterns were associated with more vitamin C. Drinking both milk and juice was associated with higher levels of calcium and vitamin C. By contrast, beverage patterns built around other caloric beverages provided the most added sugar, the consumption of which sharply increased with age.

## Discussion

Stratifying NHANES participants by beverage drinking patterns is less common than exploring beverage consumption by gender, age group, or race/ethnicity [22–26]. The present analyses assigned children into four categories: milk drinkers, 100% juice drinkers, milk and 100% juice drinkers, and drinkers of other caloric beverages. For most children, other caloric beverages were the overwhelming beverage of choice; the milk and/or 100% juice drinking patterns were relatively infrequent. Furthermore, the consumption of other caloric beverages sharply increased with age, displacing both milk and 100% juice.

HEI 2010 scores for milk drinkers and 100% juice drinkers were comparable. Milk drinkers consumed more dairy products than did juice drinkers; juice drinkers consumed more total fruit and comparable amounts of whole fruit. Milk drinking did not reduce consumption of yogurt and cheese; juice drinking did not cut into the eating of whole fruit. Diets of milk drinkers and juice drinkers did not differ in terms of energy, total or added sugars, fiber, or vitamin E. Diets of milk drinkers were higher in calcium, potassium, vitamin A and vitamin D than those of juice drinkers. Diets of juice drinkers were higher in vitamin C than those of milk drinkers.

Children rarely limit their drinking choices to a single beverage. The present analyses identified children whose drinking patterns were built around milk and 100% juice, with smaller amounts of other caloric beverages. That group had diets that were close to the dietary recommendations for both dairy (2.85 servings) and for total fruit (2.0 servings). This composite beverage pattern featuring both milk and 100% juice was associated with higher intakes of calcium, potassium, vitamin A and vitamin D along with vitamin C and with the highest HEI 2010 scores. Importantly, drinkers of milk and 100% juice consumed amounts of 100% juice that were close to AAP guidelines: 250 g for 4-8 y, 270 g for 9–13 y and 386 g for 14-19 y. The 100% juice drinkers had energy intakes that were actually lower when compared to drinkers of other beverages [6].

It should be noted that the milk pattern was strongly age-dependent; milk consumption typically decreases with age. The juice pattern showed less of an age dependence; typically 100% apple juice consumed in childhood is replaced by more citrus juices in adolescence. However, both patterns were vulnerable to a dramatic age-related increase in other beverages, with substantial amounts of added sugars.

The current dietary recommendations are for older children and teens to limit fruit juice to only one of the recommended 2 to 2 ½ cups of fruit servings per day. In the present analysis, the consumption of more than 2 fruit servings per day was only observed among exclusive or predominant 100% juice drinkers. There was no evidence in the present cross-sectional data that juice drinkers ate less whole fruit. In past studies, a combination of whole fruit and 100% juice was an effective way to address fruit shortfall, without increasing diet cost [27].

The current dietary recommendations are to replace whole milk with low fat or skim starting at age 2 y. It should be noted that milk drinking patterns follow a socioeconomic gradient. More whole milk is consumed by groups of lower socioeconomic status (SES); higher SES groups drink more low-fat and nonfat milk [19]. Lower SES groups also drink more 100% fruit juice, whereas higher SES groups eat more whole fruit. Higher SES groups drink more plain water (bottled and tap) [28]. Linking the consumption of selected beverages with health outcomes may not have adequately accounted for all the potential (and mostly unobserved) socioeconomic determinants of body weight and health [8].

The study had limitations. First, the data were based on self-report, including proxy report for younger children. However, that is a characteristic of the NHANES database, still the basis for food and nutrition policy in the US. Second, a single 24-h recall may not be sufficient to separate regular consumers from non-consumers; the database only provides beverage patterns on that particular day [21]. Third, the algorithm to identify predominant consumption of milk, 100% fruit juice or both was based on weight, as opposed to calories. However, most beverages other than diet beverages or skim milk, are of comparable energy density (< 1 kcal/g). Finally, the HEI 2010 score, technically a measure of compliance with the 2010 Dietary Guidelines, may not capture the multiple components of a healthy diet.

## Conclusions

In summary, beverage patterns built around milk and/or 100% juice were relatively rare. Most US children drank other caloric beverages. Yet beverage drinking patterns built around milk and 100% juice were associated with better dietary choices and highest quality diets. Dietary intervention strategies ought to promote rather than restrict milk and 100% juice. Based on the current analyses, milk and 100% juice, along with plain, drinking water should be the main beverages of choice.

## Additional file

Additional file 1: **Table S1.** Common varieties by beverage category. (DOCX 15 kb)

## Abbreviations

AAP: American Academy of Pediatrics
HEI: Healthy Eating Index
NHANES: National Health and Nutrition Examination Survey
