# Supplementary material for: Beverage consumption patterns among 4–19 y old children in 2009–14 NHANES show that the milk and 100% juice pattern is associated with better diets
Source: Nutr J. 2018 May 24;17:54. doi: 10.1186/s12937-018-0363-9 (PMC5968613; doi:10.1186/s12937-018-0363-9)
Supplement: Supplementary file 1 — Table S1. Common varieties by beverage category. (DOCX 15 kb) [file 12937_2018_363_MOESM1_ESM.docx]

**Supplemental Table A**. Common varieties by beverage category

| Beverage type | Three most common varieties reported |
| --- | --- |
|  |  |
| Milk and milk beverages | Whole milk, 2% milk, 1% milk |
| Citrus juices | Orange juice (canned/bottled), orange juice (w/calcium), orange juice (NFS^1^) |
| Apple juices | Apple juice, fruit juice blend, fruit juice (NFS^1^) |
| Other non-citrus juices | Grape juice, pineapple juice, prune juice |
| Soda (regular & diet) | Regular cola, regular fruit-flavored soft drink (without caffeine), sugar-free cola |
| Fruit drinks (regular & diet) | Fruit juice drink, fruit flavored drink (made from powder), fruit juice drink (w/vitamin C) |
| Sports and energy drinks (regular & diet) | Gatorade, Powerade, Red Bull (regular) |
| Vegetable juices | Tomato and vegetable juice (mostly tomato), tomato juice, carrot juice |
| Water (bottled & tap) | Tap water, bottled water |
| Flavored & enhanced water | Carbonated water (unsweetened), Glaceau water, sweetened carbonated water (e.g., tonic) |
| Alcoholic beverages | Beer, lite beer, red wine |
| Coffee | Regular coffee (from ground), regular coffee (from instant), decaffeinated coffee (from ground) |
| Tea | Unsweetened tea, pre-sweetened tea, tea NFS^1^ presweetened with sugar |
| Meal replacement beverages | High protein meal replacement, ready-to-drink meal supplement/replacement, Ensure Liquid Nutrition |
|  |  |
| Regular soda | Sugar-free cola, sugar-free cola (decaffeinated), sugar-free fruit-flavored soft drink (without caffeine), caffeinated sugar-free fruit-flavored soft drink |
| Diet (low-calorie) soda | Sugar-free cola, sugar-free cola (decaffeinated), sugar-free fruit-flavored soft drink (without caffeine) |
| Regular fruit drinks | Fruit juice drink, fruit flavored drink (made from powder), fruit juice drink (w/vitamin C) |
| Diet (low-calorie) fruit drinks | Low-calorie fruit flavored drink (powder), reduced sugar fruit juice drink (w/vitamin E), low-calorie fruit juice drink (w/vitamin C) |
| . |  |

^1^ NFS = Not further specified.
